# Hemorrhagic Colloid Cyst Presenting with Acute Hydrocephaly

**DOI:** 10.1155/2017/2978080

**Published:** 2017-01-22

**Authors:** Reza Akhavan, Behrouz Zandi, Masoud Pezeshki-Rad, Donya Farrokh, Bita Abbasi

**Affiliations:** Faculty of Medicine, Mashhad University of Medical Sciences, Mashhad, Iran

## Abstract

Colloid cysts are benign slow-growing cystic lesions located on the roof of the third ventricle that usually present with symptoms related to gradual rise of intracranial pressure. They mostly remain asymptomatic and sometimes grow progressively and cause diverse symptoms associated with increased intracranial pressure such as headache, diplopia, and sixth cranial nerve palsy. Here we report a 47-year-old female who presented to the emergency department with acute severe headache and nausea/vomiting. On MRI examination acute hydrocephaly due to hemorrhagic colloid cyst was detected. Acute hemorrhage in colloid cysts is extremely rare and may present with symptoms of acute increase in the intracranial pressure. Intracystic hemorrhage is very rarely reported as a complication of colloid cyst presenting with paroxysmal symptoms of acute hydrocephaly.

## 1. Introduction

Colloid cysts are benign embryologic remnants that are located on the roof of the third ventricle. These tumors grow slowly and may become symptomatic in third to fifth decades of life [1, 2]. The condition presents with various clinical symptoms associated with gradual increase in intracranial pressure. Acute hydrocephaly occurs uncommonly and with uncertain etiologies. Acute hemorrhage in colloid cyst is extremely rare and may present with acute symptoms and even sudden death [3, 4]. Here we report a case of acute hemorrhage in colloid cyst.

## 2. Case Presentation

**Figure 1:**
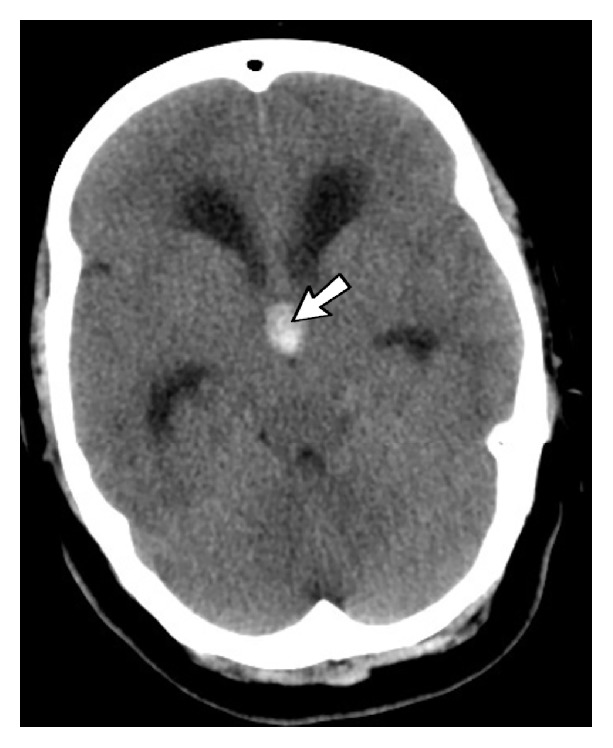
Axial noncontrast brain CT scan shows a hyperdense mass (arrow) at the location of interventricular foramen of Monro. Frontal and temporal horns of lateral ventricles are visible and enlarged.

**Figure 2:**
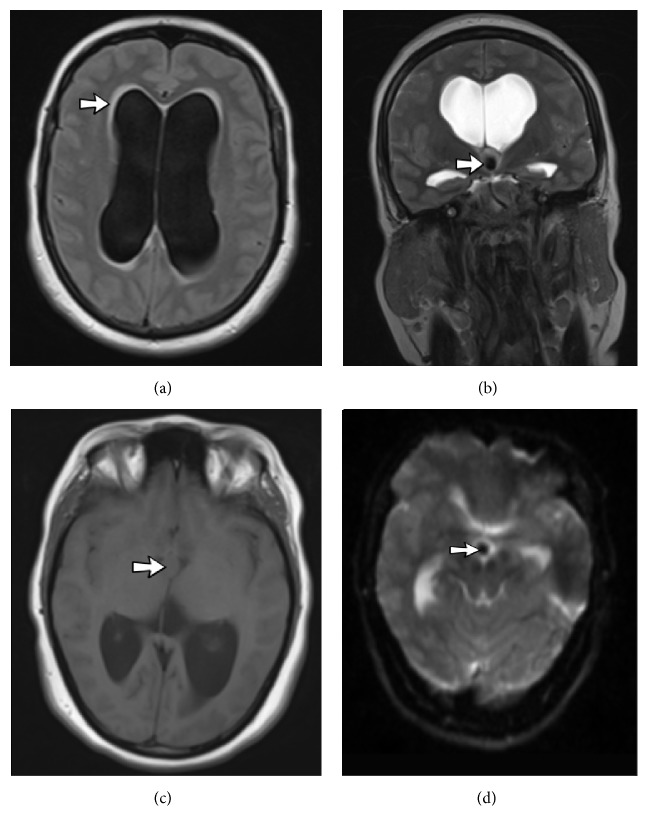
Brain MRI. Axial FLAIR image (a) shows enlargement of lateral ventricles. The hyperintense rim around the ventricles represents interstitial edema secondary to acute hydrocephaly. Coronal T2-weighted image (b) reveals a round hypointense lesion at the location of interventricular foramen of Monro (arrow in (b)). The lesion (arrow in (c)) is isointense in the axial T1-weighted image (c). The size of the lesion is exaggerated in the b0 sequence of DWI image (d); a finding that suggests hemorrhage (arrow in (d)).

The patient was a 47-year-old female who presented to the emergency department with acute headache and nausea/vomiting. She did not mention any previous history of severe headache or vomiting. On the neurologic examination papilledema was noted. There were no focal neurological deficits. She was dizzy but conscious and her Glasgow coma scale (GCS) score was 13. Computed tomography (CT) scan of the brain revealed enlargement of lateral ventricles and a well-circumscribed hyperdense mass on the roof of the third ventricle at the location of foramen of Monro (Figure 1). On MRI examination, a hyperintense rim was seen around lateral ventricle on FLAIR sequence that was compatible with interstitial edema and acute nature of hydrocephaly (Figure 2(a)). A T2-hypointese (Figure 2(b)), T1-isointense mass was seen on the roof of the third ventricle (Figure 2(c)). The characteristic location and MRI signal intensity were consistent with colloid cyst. Blooming artifact was visible on susceptibility weighted sequence (Figure 2(d)) and suggested the hemorrhagic nature of the lesion and explained the occurrence of acute hydrocephaly.

The patient underwent ventriculostomy followed by craniotomy and tumor resection and experienced an uneventful recovery. Histological examination confirmed the diagnosis of hemorrhagic colloid cyst.

## 3. Discussion

Colloid cysts are benign tumors of brain that rise from endodermal embryologic remnants and are classically located on the roof of the third ventricle [1, 2]. The lesions mostly remain asymptomatic and sometimes grow progressively and cause diverse symptoms associated with increased intracranial pressure such as headache, diplopia, and sixth cranial nerve palsy. Acute paroxysmal symptoms might occasionally follow acute hydrocephaly. The exact pathophysiology of acute hydrocephaly is not well understood. Some authors have suggested changes in intracranial pressure (lumbar puncture, etc.) as a possible cause of acute hydrocephaly [5]. Intracystic hemorrhage is very rarely reported as a complication of colloid cyst presenting with paroxysmal symptoms of acute hydrocephaly [3, 4]. Although we assume acute hemorrhage to be a possible cause of paroxysmal symptoms in our case, there is no way to prove it and the exact cause of acute hydrocephaly in this patient (as in many other cases with the same pathology) is not known for sure.

Approximately two-thirds of the cysts are hyperdense on computed tomography due to their high protein content; the remaining third appears isodense [3]. On MRI examination, the lesion's signal intensity is dependent of its protein content, being mostly hypointense on T2-weighted and iso- to hyperintense on T1-weighted images. The blooming artifact seen in susceptibility weighted sequence suggested the hemorrhagic nature of the cyst in our patient, an assumption that was confirmed in histological examination.
